# Synthesis of 4-Amino-*N*-[2 (diethylamino)Ethyl]Benzamide Tetraphenylborate Ion-Associate Complex: Characterization, Antibacterial and Computational Study

**DOI:** 10.3390/molecules28052256

**Published:** 2023-02-28

**Authors:** Gamal A. E. Mostafa, Ahmed H. Bakheit, Mohamed H. Al-Agamy, Rashad Al-Salahi, Essam A. Ali, Haitham Alrabiah

**Affiliations:** 1 Department of Pharmaceutical Chemistry, College of Pharmacy, King Saud University, P.O. Box 2457, Riyadh 11451, Saudi Arabia; 2 Department of Pharmaceutics, College of Pharmacy, King Saud University, P.O. Box 2457, Riyadh 11451, Saudi Arabia

**Keywords:** procainamide, tetraphenyl borate, ion-associate complex, characterization, antibacterial activity, computational study

## Abstract

The 4-amino-N-[2 (diethylamino) ethyl] benzamide (procainamide)-tetraphenylborate complex was synthesized by reacting sodium tetraphenyl borate with 4-amino-N-[2 (diethylamino) ethyl] benzamide, chloride salt, and procainamide in deionized water at room temperature through an ion-associate reaction (green chemistry) at room temperature, and characterized by several physicochemical methods. The formation of ion-associate complex between bio-active molecules and/or organic molecules is crucial to comprehending the relationships between bioactive molecules and receptor interactions. The solid complex was characterized by infrared spectra, NMR, elemental analysis, and mass spectrometry, indicating the formation of ion-associate or ion-pair complex. The complex under study was examined for antibacterial activity. The ground state electronic characteristics of the S1 and S2 complex configurations were computed using the density functional theory (DFT) approach, using B3LYP level 6-311 G(d,p) basis sets. R^2^ = 0.9765 and 0.9556, respectively, indicate a strong correlation between the observed and theoretical ^1^H-NMR, and the relative error of vibrational frequencies for both configurations was acceptable, as well. HOMO and LUMO frontier molecular orbitals and molecular electrostatics using the optimized were used to obtain a potential map of the chemical. The *n* → π* UV absorption peak of the UV cutoff edge was detected for both configurations of the complex. Spectroscopic methods were structures used to characterize the structure (FT-IR and 1HNMR). In the ground state, DFT/B3LYP/6-311G(d,p) basis sets were used to determine the electrical and geometric properties of the S1 and S2 configurations of the title complex. Comparing the observed and calculated values for the S1 and S2 forms, the HOMO-LUMO energy gap of compounds was 3182 and 3231 eV, respectively. The small energy gap between HOMO and LUMO indicated that the compound was stable. In addition, the MEP reveals that positive potential sites were around the PR molecule, whereas negative potential sites were surrounding the TPB site of atoms. The UV absorption of both arrangements is comparable to the experimental UV spectrum.

## 1. Introduction

Due to their unique chemical and physical features and wide variety of potential uses, ionic liquids at room temperature have become one of the most investigated topics for both theoretical and industrial purposes [1,2]. Ionic liquids, especially at room temperature, have provided a potent green chemistry strategy for a variety of industrially relevant chemical processes [3]. The degree of ionic association within each ionic liquid is expected to have a major effect on many of its properties. In addition to the constituent cations and anion of the ionic liquid, the solvent medium (in which the ionic liquid is dissolved) and the particular ionic density profile also have a significant impact on the strength of the ionic connection. Water, in particular, is important in ionic liquid applications. It has been found that the majority of ionic liquids are very hygroscopic, meaning that even a small amount of water can significantly alter their physical behavior [4]. Unsurprisingly, there is a great deal of interest today in learning everything there is to know about ion–ion and ion–water interactions [5,6,7,8].

Ion-pairs are used to make new, controlled-release pharmaceutical formulations, especially for peptides [3,9,10]. One of the key benefits in this situation is that the active ingredient does not undergo structural changes following ion pairing, which preserves its pharmaco-toxicological profile. Drugs can be made to be more stable [11] and bioavailable [12,13]. In a number of kinetic studies, the production of ions pairs was proposed as a possible method for pharmacological medications to be absorbed [14,15]. Ion-pair equilibrium has important applications in biochemistry, including studies on DNA stability in diverse matrices [15], protein identification [16], and the manufacture of ion-pair receptors based on biological models [17]. Isolation, identification, and quantification of some compounds of biomedical interest are all capabilities of ion-pair-based assay methods [18]. Gravimetric [19], electrometric [20,21,22], spectrophotometric [23,24], and chromatographic techniques based on ion-pair production have all been developed over time [25,26]. Procainamide-tetraphenylborate ion-associate complex was used as a potentiometric sensor to quantify procainamide in the drug’s formulation and to spike urine [27].

Procainamide hydrochloride, a class 1A antiarrhythmic medication, is used to treat cardiac arrhythmia in patients with heart disease [27]. In contrast, this study focuses on experimental and theoretical studies of the ion-pair formation in water, as an ionic liquid at ambient conditions, as a green method. This investigation aims to synthesize and characterize antibacterial activity and theoretically study the procainamide-tetraphenyl borate ion-associate or ion-pair complex. On the other hand, the complex under study had its biological activity tested for its antibacterial activity.

## 2. Results and Discussion

### 2.1. Chemistry

**Scheme 1:**
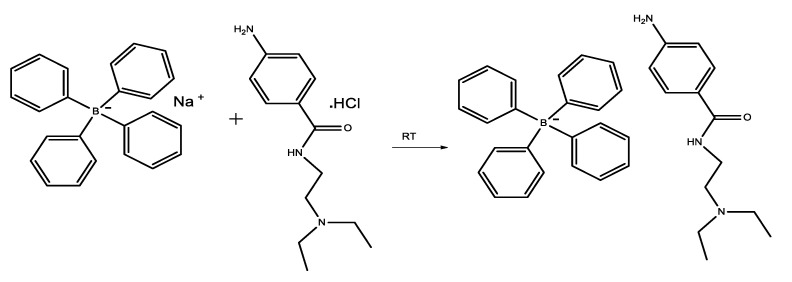
The synthetic pathway of the tetraphenylborate salt of procainamide.

The title compound, 4-amino-N-[2-(diethylamino) ethyl] benzamide tetraphenylborate, was produced in 85% of the reactions between sodium tetraphenyl borate and 4-amino-N-[2-(diethylamino) ethyl] benzamide hydrochloride (procainamide hydrochloride) in deionized water at room temperature (Scheme 1).

The ^1^H NMR spectrum of procainamide-tetraphenyl borate complex is characterized by the triplet signal of the N-(CH_2_)2 (CH_3_)2 protons at *δ* 1.22 ppm, triplet signals protons at *δ* 3.20 and 3.34 ppm for the (-N-CH_2_-CH_2_-N-) protons, quadrat signal at 3.54 ppm for the N-(CH_2_)2 (CH_3_)2 protons, and a singlet signal for NH_2_ at 5.72 ppm (Figure S1 in the Supplementary Materials). Meanwhile, the ^13^C NMR spectrum of the target complex revealed the resonance peaks at *δ* 8.87, 34.87, 47.20, and 51.06 ppm for N-(CH_2_)2 (CH_3_)2, (-N-CH_2_-CH_2_-N-), N-(CH_2_)2 (CH_3_)2, and (-N-CH_2_-CH_2_-N-) (Figure S2), respectively. The aromatic protons for both procainamide and tetraphenylborate rings appeared in the region 6.57–7.56 ppm, whereas the resonance ^13^C NMR peaks appeared between 112.77–164.27, and the carbon for amide groups revealed at 167.66 ppm.

The IR spectrum of the target was characterized by a strong absorption band at 1633 cm ^−1^ for the carbonyl group and weak absorption peaks at 3230–3469 cm ^−1^, assignable for amine groups (Figure S3). The ESI-MS revealed a peak at (*m*/*z*) 336.49 for [M]^+^, in addition to a negative scan at (*m*/*z*) 319.2 for [M-] (Figure S4). In accordance with the theoretical values of C 77.89%, H 7.24%, and N 7.36%, the elemental analysis data reveal C 78.33%, H 7.73%, and N 7.47%.

### 2.2. Electronic Absorption Spectra

**Figure 1:**
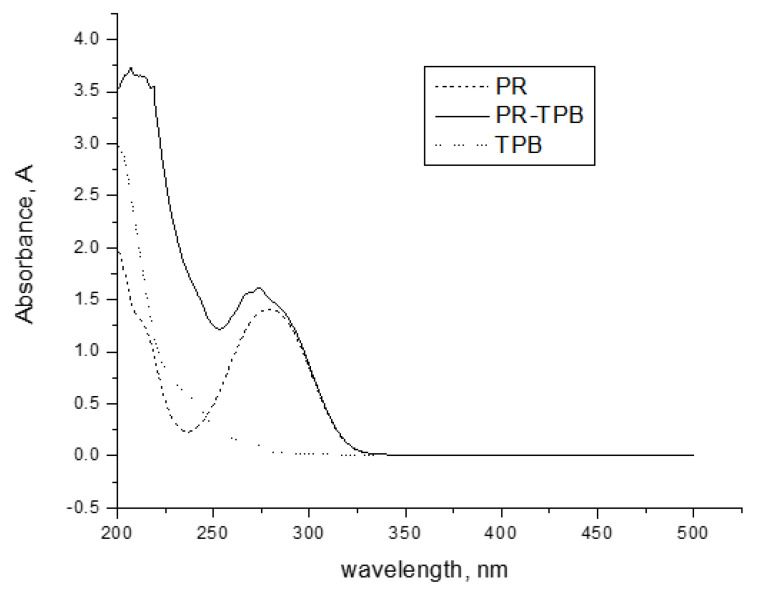
Absorption spectra of procainamide, sodium tetraphenyl borate, and procainamide-tetraphenyl borate (0.0001M, each).

The spectrophotometric study was used to characterize the development of ion-association complex. Figure 1 illustrates how the ion-associate complex formation was related to the complex absorption spectra. Sodium tetraphenylborate displayed a peak at 200 nm, and procainamide displayed peaks at 200 and 279 nm. At 274 nm and 210 nm for the procainamide-tetraphenyl borate ion-associate, respectively, there were no absorption maxima for procainamide and/or tetraphenylborate (Figure 1). Figure 1 shows the UV spectra of the formed ion-associate with a maximum wavelength of 210 nm and a shift in the greatest absorption when compared to the compounds procainamide and tetraphenyl borate.

### 2.3. Antimicrobial Activities

The results of the zone of inhibition and MIC are shown in Table 1. The results revealed that the PR-TPB complex has good activity against both “Gram-positive bacteria (*S. aureus* ATCC 29,213 and *B. subtilis* ATCC 10400) and yeast, including *Candida albicans* ATCC 10231”. The PR-TPB compound has weak activity against Gram-negative bacteria (*E. coli* ATCC 10,418 and *Ps. aeruginosa* ATCC 27853).

### 2.4. Computational Study

#### 2.4.1. DFT Optimization Investigation

**Figure 2:**
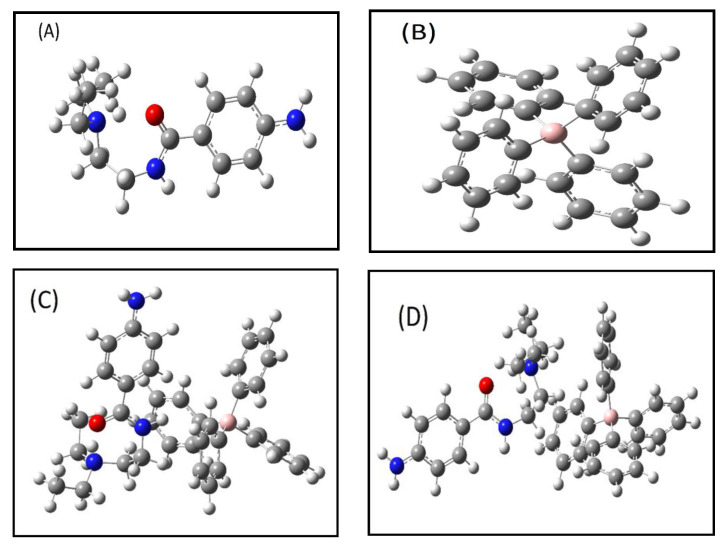
B3LYP/6-311G(d,p) optimized geometries of the molecules: (**A**) procainamide cation, (**B**) tetraphenylborate anion, (**C**,**D**) procainamide cation, and tetraphenylborate anion salt.

For optimizing the geometries of the procainamide cation, tetraphenylborate anion, and their salt in the gas phase, the B3LYP density functional theory-based function with 6-311G(d,p) basis sets was found to be the most suitable (Figure 2) [28,29]. We were unable to compare the calculated bond lengths and bond angles with actual data since the crystal structure of the chemical in question is not yet accessible. Figure 2 depicts the geometries that best characterized the energetically ideal ion-pair combinations, their thermodynamic stability, and bond interactions. In addition, the ideal structure of the procainamide cation is shown in Figure 2A, while the optimal structures of the tetraphenylborate anion are shown in Figure 2B. In addition, the salt-optimized geometries are shown in Figure 2C. All of these geometries represent the local minima of surface potential energy.

#### 2.4.2. Interaction Energies (IE)

The DFT technique at the B3LYP level using the 6–311G(d,p) basis set has been frequently utilized to determine the most stable configurations and binding energies of structures. Therefore, the complexation energy and BSSE energy of the procainamide cation, tetraphenylborate anion, and two salts of the procainamide cation with the tetraphenylborate anion (PR–TPB) (molar ratio 1:1) were computed and presented in ion Table 2. The salts of the procainamide cation with the tetraphenylborate anion in the gas phase have complexation energy and (corrected) and BSSE energy of–99.13 kcal/mole and 0.00922, respectively, while another salt with the same ratio has complexation energy and (corrected) and BSSE energy of–94.64 kcal/mole and 0.00645, respectively. Consequently, these results of the complexation energy for both salts reveal a low energy of formation, indicating that both complexes were stable. In addition, the complexation energy of these complexes is negative, showing that they are easy to produce spontaneously and that the outcome is in accordance with the experimental procedure.

#### 2.4.3. Electronic Absorption Spectrum Analysis (UV-Vis Spectroscopy Analysis)

**Figure 3:**
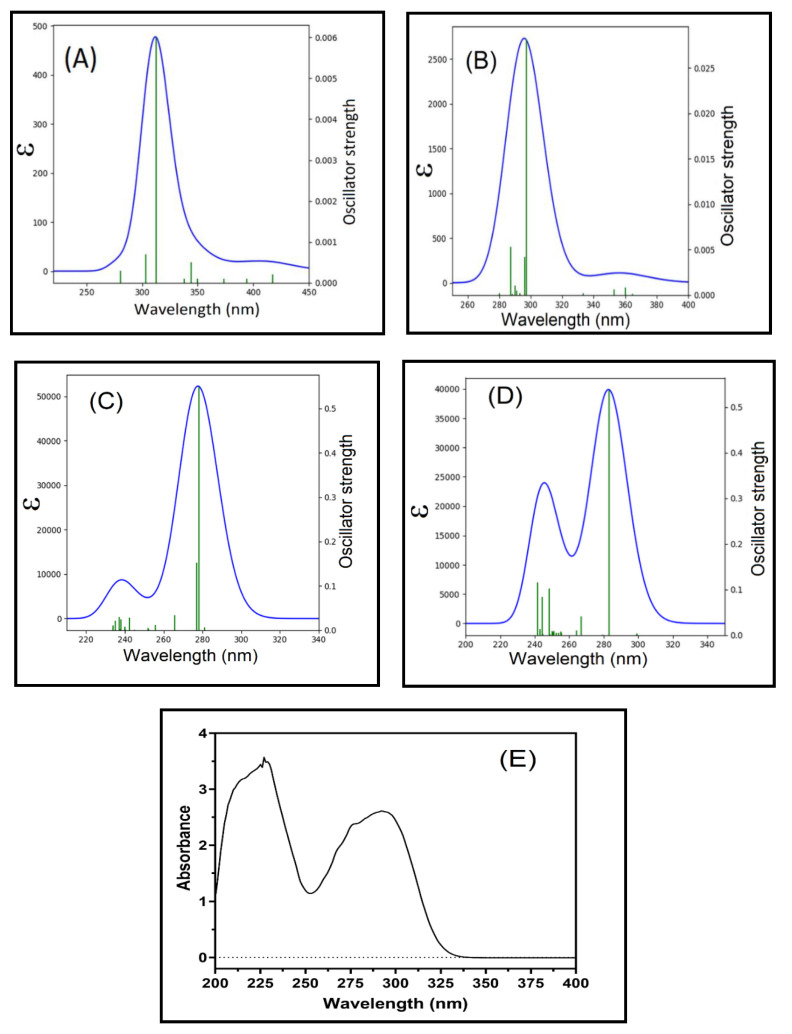
(**A**–**C**) TD-DFT calculated absorption spectra in the gas phase and water solvent for complexes conformation S1 (**A**,**C**) and S2 (**B**,**D**), respectively. (**E**) Experimental absorption spectrum of the title complex in water solvents at room temperature.

The electronic spectrum analysis of the aforementioned structure was conducted in both the gas and water solvent phases using the time-dependent-DFT (TD-DFT) IEFPCM model at the B3LYP/6-311G(d,p) level theory on the optimized ground state geometry. Nonetheless, the acquired absorption spectra were investigated at room temperature in aqueous solvents with a 40ppm concentration, and the resulting plots are presented in Figure 3.

At room temperature, the experimental maximum wavelength (*λ_max_*) and absorbance were determined, and the data are given in Table 3. The dipole moment (*μ*, D), main absorption energy (E), oscillator strength (*f*), maximum wavelength (max), electronic transition of excitation energy, and atomic orbital contribution were computed in solvent and gas phases, and the corresponding data are shown in Table 3. The measured absorption spectra of the title chemical were obtained in aqueous solvents due to the solubility of the product complexes in water. The molecule exhibited the high-intensity band at 292, and 225 nm in the water solvent, which may be attributed to the n → π* electronic transition, as shown in Figure 3E. The molecular orbitals of auxochrome as (NH_2_) or (C=O), exocyclic, nitrogen lone pairs and the conjugated π-bond of the phenyl of the tetraphenylborate moiety caused this electronic transition. The complexes exhibited a negative absorption solvatochromism with water solvent, indicating that the absorption moved to the blue with the polar solvent. The transitional energy was observed in the water solvent more than in the gas phase [30,31,32,33].

In Figure 3A–C, the computation was, therefore, conducted using the TD-DFT-IEP-PCM model. In the gaseous state, strong absorption bands were found at 312 nm (*f* = 0.006) for structure S1 and 297 nm (*f* = 0.0287) for structure S2. The computation was, therefore, continued using the “TD-DFT-IEP-PCM” model. In the gaseous state, a high absorption band was found at 312 nm (*f* = 0.006) and 297 nm (0.0282) for the estimated structures S1 and S2, respectively, corresponding to the excitation of electrons that occurred during the HOMO-to-LUMO transition (Table 3).

**Figure 4:**
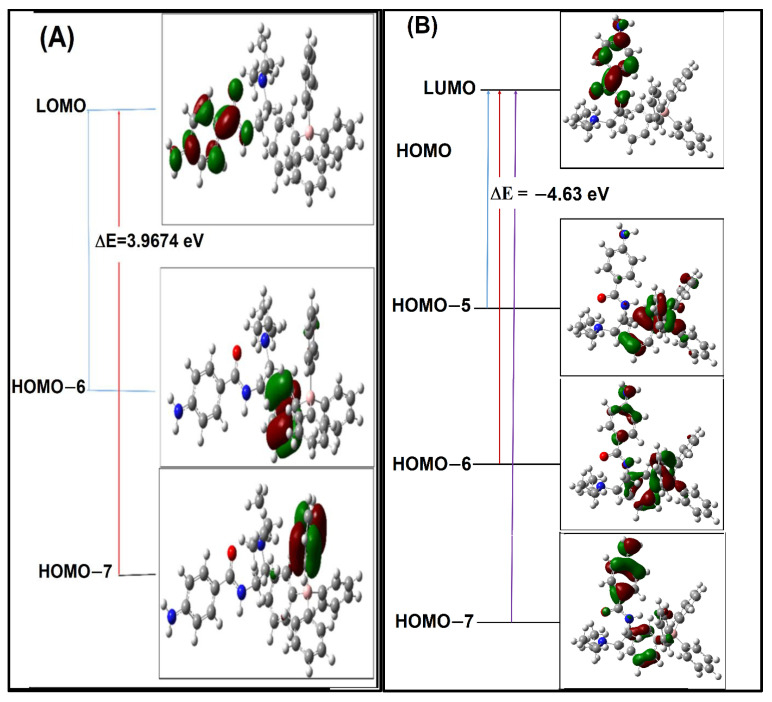
Electronic absorption in the gas phase is facilitated by band gaps (ΔE) and frontier molecular orbitals in both (**A**) complex S1 and (**B**) complex S2 (E).

The distribution of the energy levels and electronic transitions of molecular orbitals in the complexes was in both the solution and gas phases. The frontier MO analysis in calculating the energies and the characteristics of frontier MOs implies that these orbitals are the most viable locations of exchange density, leading to orbital contact. There was a little difference between the energy gap between the gas and solvent phases. The intramolecular charge transfer (S_0_ → S_1_) transition corresponds to the estimated energy gap (E = HOMO-LUMO) values of 4.4566 and 4.3795 eV for complex S1 and S2 in the solvent phase, respectively (Figure S1). Due to the various polarities and dielectric constants of the complexes, it is expected that water might stabilize them by increasing the energy gap between their HOMOs and LUMOs. Additionally, the energy gap was changed by the polarity of the solvent. In compound S1, bonding was detected on the tetraphenylborate moiety phenyl ring (Figure 4A), based on the H-6 and H-7 orbitals. Figure 4A identifies non-bonding (n-type) nature orbitals on the nitrogen atom of the exocyclic amino group, based on the LUMO orbital. In contrast, the H-5, H-6, and H-7 orbitals were centered on the bonding of the tetraphenylborate moiety and, in the PR moiety, in complex S2. Figure 4B identifies nature orbitals on the nitrogen atom of the exocyclic amino group, whereas LUMO orbitals are focused on non-bonding (n-type) atoms. The results indicate that the water solvent increases the transition probability (n → π*) of the current molecule. The second complex conformation was the more advantageous of the two. There is a strong connection between the TD-DFT and experimental absorption spectra of the second complex conformation.

#### 2.4.4. Vibrational Frequencies in IR-Spectrum

The calculation of the vibrational frequencies, using VEDA 4 software [34,35], in the IR-spectrum of the ionic coupling of the PR with the TPB forms complex with two configurations were carried out by using the “B3LYP function with the 6-311 G(d,p)” basis set. The chosen characteristic vibrational band values, along with the observed values, are presented in Table 4. In the literature, the amine N-H stretching frequency shows at 3500–3300 cm^–1^ in the infrared spectra, and this band appeared with mild absorption at 3230–3469 cm^–1^ for the complex. Meanwhile, the computed IR spectra for both configurations were 3589.56 cm^−1^ and 3552.23 cm^−1^ for S1 and S2 of the complex, respectively. The computed vibrational value agreed with the observed value. The most significant vibrational stretching absorbance was discovered at 1633 cm^−1^ due to the amide C=O stretch group of the 4-Aminobenzamide ring, with the calculated values being 1657 cm^−1^ and 1618.76 cm^−1^ for the S1 and S2 configurations, respectively. The strong stretching band seen for the amide C=O group may be a result of electrical processes (unshared electron pairs on the O and N atoms) and intermolecular hydrogen bonding involving neighboring atoms.

#### 2.4.5. Benchmark of ^1^H NMR Chemical Shifts

**Figure 5:**
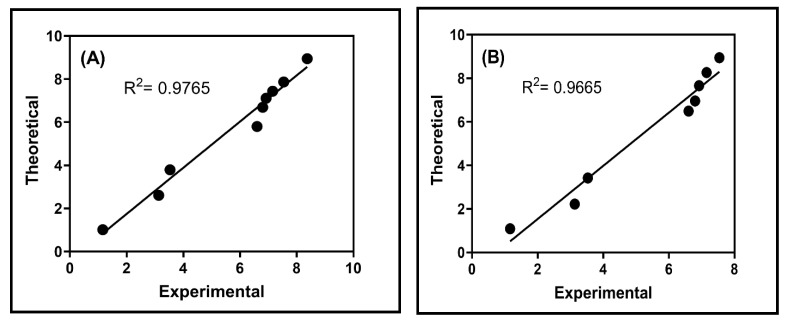
Correlation studies between observed and calculated (**A**,**B**) ^1^H-NMR of conformation S1 and S2 of complex, respectively.

The calculated ^1^H NMR spectra of the complex with both configurations were analyzed by using “B3LYP/6-31+ G(d,p) in DMSO, and the chemical shift (^1^H NMR)” values are presented in Figure 5 and Table 5. The computed and observed chemical shifts for both configurations are very similar. Except for the nitrogen atom of the amide group bonding with H58 protons, and CH in the terminal chain of PR. The calculated deviation was observed due to the van der Waals interactions with TPB phenyl rings and was given in Table 5.

#### 2.4.6. Non-Covalent Interaction (NCI) Index

**Figure 6:**
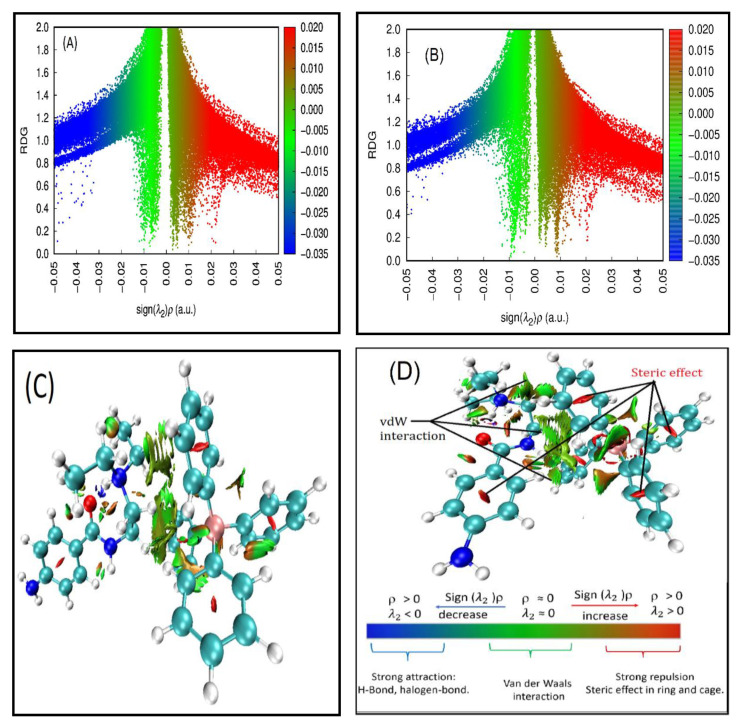
Standard NCI index representations and RDG scatter plots (**A**,**B**) at the B3LYP/6-311G level. The RDG cut-off is sign(λ_2_)ρ = 0.5 a.u, and the color scale is -0.030 to 0.02 a.u, (blue, green, and red surfaces imply attracting, van der Waals, and repulsive interactions, respectively) for both S1 and S2 configurations, and (**C**,**D**) isosurface maps for both S1 and S2 configurations, respectively.

Similar to the AIM approach, the reduced density gradient (RDG) is an efficient methodology for accounting for non-covalent interactions. RDG scatter plots and NCI plots can be used to visualize the non-covalent interactions between molecules [36,37]. In this approach, RDG is plotted against electron density multiplied by the sign of the second eigenvalue (sign (2)) [37], and weak intermolecular and intramolecular interactions are displayed in Figure 5. The green and red spikes on the positive scale of sign (2) represent van der Waals interactions and steric repulsions, respectively. The non-covalent interactions of the investigated complex are depicted in Figure 6A,B (0.025 a.u.). For the first predicted structure, the green-colored RDG region corresponds to the bonds C2⋯H64, H19⋯H64, C25⋯H64, C4⋯H60, C5⋯H61, C3⋯H75, C27⋯H67, and C31⋯H74. In contrast, the second predicted structure consists of the atoms N12⋯C41, H8⋯H44, H18⋯C47, H26⋯C43, H15⋯C52, H8⋯H57, and H13⋯C53.

In addition, the NCI plot is utilized in Figure 6C,D to illustrate van der Waals interactions and steric effects of the investigated complex. Notably, the complex molecules contain aromatic moieties that appear to be engaged in the van der Waals contact with the strong RDG spikes seen in green between the molecules in Figure 6C,D. In addition, the orientation of the PR to TPB differed between the two configurations; in the S1 configuration, the amine group in the chain terminal was oriented to TPB and interacted with TPB, whereas in the S2 configuration, the NH of the amide group in the middle of the molecule was oriented to TPB and interacted with the TBP ion. In the S1 configuration, there were eight van der Waals interactions between TPB and PR ions, but in the S2 configuration, there were only seven van der Waals interactions (Table 6) [31].

#### 2.4.7. Quantum Theory of Atoms in Molecules (QTAIM)

**Figure 7:**
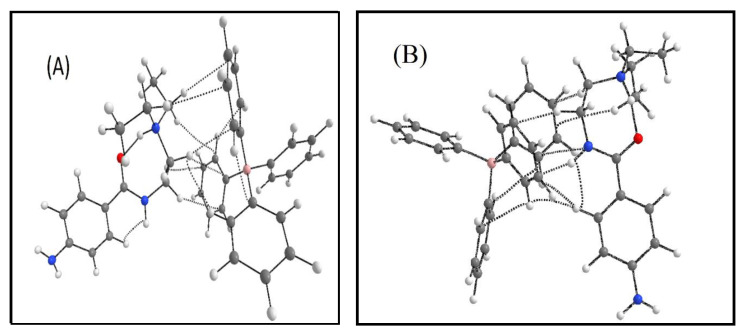
Topological atoms in molecules (AIM) graph of both PR-TPB complexes, configuration for: (A) complex S1 and (B) complex S2.

The late R. F. W. Bader came up with the Quantum Theory of Atoms in Molecules, which has been used a lot in research to figure out the topological features of different kinds of hole interactions [31,36,38]. Using the DFT:B3LYP/6-311G(d,p) basis set, AIM analysis was performed on all compounds to acquire a better understanding of the noncovalent HO bonds. Table 6 and Figure 7 illustrate the categorization of intermolecular bonds in both anticipated salt complex structures. The bond critical points (BCPs) for the first salt structure, S1, were determined to be at positions 1, 19, 30, 72, 73, 76, 77, and 90. In contrast, the BCP for the second salt structure, S2, was calculated to be 14, 44, 45, 48, 58, 61, and 62, according to Koch and Popelier [39,40,41], using the results of the topological study of the electron density for both salt complexes.

Because their Laplacian ∇^2^ρ(r) values are positive and in the range (0.0113-0.0195 a.u.), and they also have positive total energy density H(r) values and in the range (0.0113-0.0195 a.u.), they have a positive total energy density (0.0005-0.0008 a.u.), moreover, All bonds are van der Waals interactions (closed-shell interactions), as well, because the absolute potential energy density to kinetic energy density ratio |V(r)|/G(r) is less than 1 in the range (0.0917−0.154). In addition, all bonds at the BCP were classified as van der Waals interactions based on the values of ρ(r) and ∇^2^ρ(r), which are within the van der Waals interaction limit [31].

#### 2.4.8. MESP Analysis

**Figure 8:**
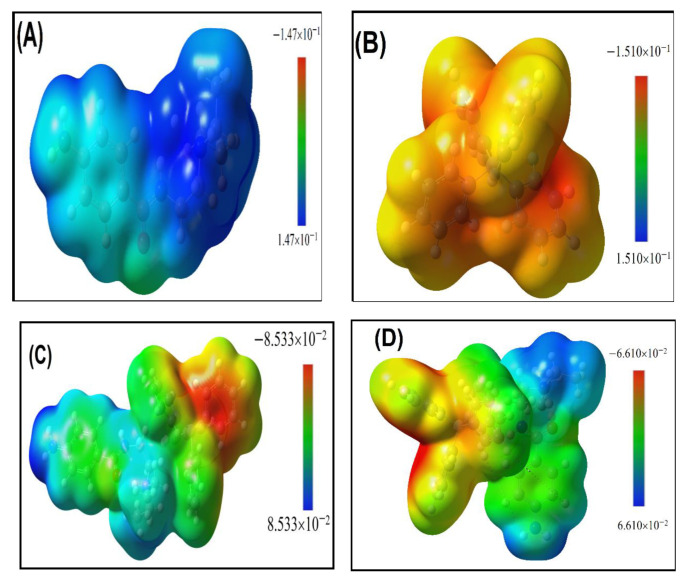
Electrophilic site study by MESP for: (**A**) procainamide cation, (**B**) tetraphenylborate anion, (**C**) S1 configuration, and (**D**) S1 configuration of complex of procainamide with tetraphenylborate, superimposed on the structure’s isodensity surface (isovalue = 0.002), which was calculated at the B3LYP/6311 G (d, p) level.

The MEP surfaces [42,43] are the most effective graphical representations of the electrostatic potential over the surface of a molecule for detecting electrophilic and nucleophilic sites. are the best graphical representations of the electrostatic potential across the surface of a molecule for the purpose of identifying the electrophilic and nucleophilic centers. The MEP surfaces are color-coded, with blue for the most positive area, red for the most negative, and green for the neutral area. In the gas phase, Figure 8 depicts the MEP maps of PR, TPB, and the configuration of both the complexes S1 and S2. Figure 8A depicts the MEP plot of the acceptor (Procainamide), which is characterized by a positive surface (blue) (with a surface map value of 0.147 au) in the chain terminal and amide (near the NH group), which is considered an electrophile. In the donor (TPB) MEP plots (Figure 8B), the negative region on the phenyl ring is predictive. Regarding the predominant negative region (red) of the TPB (with a surface map value of –0.151 au), it is referred to as an n-donor (nucleophile). The MEP plot of the product complex between the donor and acceptor follows. As a result of charge transfer from the donor to the acceptor in the S1 configuration, the surface map values of the donor decrease (–0.0855 au), while those of the acceptor rise (0.0855 au). Accordingly, regarding the charge transfer from the donor to the acceptor during the S2 configuration, the donor’s surface map values decrease by –0.0661 au, while the acceptor’s values increase by 0.0661 au. Consequently, the ESP map surfaces are in great accordance with the experimental findings.

### 2.5. Reactivity Descriptors

Numerous reactivity descriptors, including “ionization potential (Ip), electron affinity (A), chemical potential (μ), hardness (η), electrophilicity index (ω), and softness (σ), are estimated from the HOMO(N), HOMO (N+1), and HOMO (N-1) surfaces, providing insight into the reactivity of the chemical reactions”. Equations are used to describe these characteristics. Table 7 summarizes the electrical interactions of PR and TPB with generated charge transfer complex characteristics. The electrical properties of the PR and TPB molecules are deduced from this table. When determining a molecule’s HOMO−LUMO energies, a high E_HOMO_ indicates a good electron donor, whereas a low E_LUMO_ indicates a good electron acceptor. Because PR has a lower E_LUMO_ than TPB in the gas analysis, it is regarded as an electron acceptor; yet, because TPB has a higher E_HOMO_ than PR, it is considered an electron donor. In complex reactions, the electronic chemical potential of PR and TPB is listed in Table 7. While anion TPB, with an energy level of –5.618 eV according to Table 7, will act as strong electron-acceptor molecules during the formation of this complex, PR, with an energy level of –3.148 eV, will act as strong electron-donor molecules.

Additionally, the electrophilicity scale has compounds with electrophilicity numbers greater than 1.50 eV, which make up Group I of strong electrophiles. A second group of moderate electrophiles (group II) consists of compounds with electrophilicity values between 1.49 and 0.90 eV. Group III of marginal electrophiles consists of compounds with electrophilicity values less than 0.90 eV [44]. Therefore, since the electrophilicity of TPB is greater than that of PR, TPB is the superior electrophile and should be considered the e-acceptor, whereas PR is the e-donor.

## 3. Experimental Section

### 3.1. Materials and Instrument

Melting point (uncorrected) was determined on a Gallenkamp melting point apparatus. NMR Spectra were scanned in DMSO-d_6_ on a Brucker NMR spectrometer operating at 500 MHz for ^1^H and 125 MHz for ^13^C. Chemical shifts are expressed in δ-values (ppm) relative to TMS as an internal standard. Coupling constants (J) are expressed in Hz. D_2_O was added to confirm the exchangeable protons. Mass spectrum was measured on an Agilent Triple Quadrupole 6410 QQQ LC/MS equipped with an ESI (electrospray ionization) source. A Shimadzu 1800 UV double beam spectrophotometer with quartz cell was used, as was a PerkinElmer, PE 2400 series II CHNS/O analyzer for elemental analysis.

### 3.2. Synthesis of 4-Amino-N-[2-(Diethylamino) Ethyl] Benzamide Tetraphenylborate

To a solution of 4-amino-N-[2-(diethylamino) ethyl] benzamide hydrochloride (procainamide hydrochloride) (0.27178 g, 1 mmol) in deionized water (20 mL), a solution of sodium tetraphenylborate (0.3422 g, 1 mmol) in deionized water (20 mL) was added. The former white precipitate was filtered off, washed with cold deionized water, and dried over anhydrous CaCl_2_ white solid (yield > 85%). The melting point of the ion-associate complex was 177 °C, which is different than the reactants (185 °C and **>**310 °C) for procainamide and sodium tetraphenylborate, respectively.

IR (KBr cm^−1^): 1633 cm ^−1^ for (C=O) and 3230-3469 cm ^−1^ for NH and NH_2_. ^1^H NMR (700 MHz, DMSO_d_6_) δ: 1.22 (t, J = 7.0 Hz, 6H), 3.20 (t, J = 7.0 Hz, 2H), 3.34 (t, J = 7.0 Hz, 2H), 3.54 (q, J = 14.0 Hz, 4H), 5.72 (s, 2H, NH_2_), 6.57-8.28 (all ArH), 9.09 (s,1H, NH). ^13^C NMR δ: 8.87, 34.87, 47.20, 51.06, 112.77, 120.31, 121.68, 125.66, 129.25, 136.00, 152.75, 163.41, 163.67, 163.97, 164.27, 167.66.

### 3.3. Antifungal and Antibacterial Screening

The plate diffusion method, utilizing cups, and the calculation of the minimum inhibitory concentration (MIC) were used to screen the ion-pair complex (PR-TPB) for antimicrobial activity, using the previously described procedures [45,46].

#### 3.3.1. Cup-Plate Diffusion Method

By using the cup-plate diffusion method, the ion-associate complex was tested against “Gram-positive bacteria (Staphylococcus aureus ATCC 29213 and Bacillus subtilis ATCC 10400), Gram-negative bacteria (Escherichia coli ATCC 10418 and Pseudomonas aeruginosa ATCC 27853), and a fungus yeast (Candida albicans ATCC 10231).” Dimethyl sulfoxide (DMSO) was used to dissolve the PR-TPB complex, resulting in a final concentration of 5120 µg/mL. The quadrant streak plate technique was used to cultivate the standard ATCC strains onto tryptone soy agar in order to purify them. To achieve a turbidity comparable to 0.5 M McFarland reagent, three to five well-isolated, overnight, pure cultures of the standard strains were suspended in sterile normal saline. The inoculum was swapped into the plate Mueller–Hinton agar. The cups were then completed inside the inoculated plates. After that, 100 µL of the tested drug and 100 L of DMSO solution, which served as the negative control, were distributed into separate cups. A ketoconazole (30 µg) disk served as the standard positive control for antifungal activity, whereas an imipenem (10 µg) disk served as the standard negative control for antibacterial activity. The plates were then given a 24 h aerobic incubation period at 37 °C. Following the incubation period, the outcomes were documented by using a ruler to measure the zone of inhibition (mm) and recording the results. The antimicrobial assay was performed in triplicate, and the mean value was computed.

#### 3.3.2. Minimum Inhibitory Concentration (MIC)

The PR-TPB complex was tested against standard ATCC strains to determine its MIC using the broth dilution method. Mueller–Hinton broth was briefly poured into a sterile tube in an amount of 1 mL. There were 10 tubes. Tubes 9 and 10 were employed as positive and negative controls, respectively, for the medium’s sterility. Tube 9 served as the positive control (no tested substance) (no microorganism). The first tube received 1 mL of PR-TPB solution (5120 g/mL) and was thoroughly mixed. To create a twofold dilution, 1 mL of the first tube was then put into the second tube. The eighth tube was reached by repeating this process down. Only 1 mL of the seventh tube was wasted. With the exception of the negative control tube, all tubes received 1 mL of the modified inoculate. As positive controls for the antibacterial and antifungal, respectively, imipenem and fluconazole were utilized. For 20 h, the infected tubes were incubated at 37 °C. After the incubation time, the MIC results were manually entered.

### 3.4. Computational Details

Computational Information. In the previous several decades, DFT has evolved from a rising star to a prominent actor in computational quantum chemistry. Target molecule conformational geometries were calculated using DFT. The Gaussian 09 software package [47] was applied for all calculations. Although attempts are being made to provide more “general-purpose” capabilities, it is known that certain features are better suited to certain applications. The B3LYP functional [48,49], which employs the basis set 6-311G(d,p) [50], has been successfully used for reactivity investigations due to its advantageous balance of accuracy and computational cost. Using the conventional 6-31G(d,p)/6-311G(d,p) basis set, the initial geometry derived using traditional geometrical parameters was reduced at the DFT level, without any limitations in the potential energy surface [4]. On the optimized structure, time-dependent DFT was utilized to determine electronic absorption spectra (TD-DFT) [51]. Using a technique developed by Wolinski et al. [52,53], the ^1^H and ^13^C chemical shifts were calculated on a δ-scale with respect to the TMS. This method is referred to as the Gauge-Independent Atomic Orbital (GIAO) at B3LYP/6-31+G(d,p), and it was used to estimate the ^1^H and ^13^C NMR (DMSO solvent). As a starting point for developing the fundamental structures of complex formation ion pairs, the geometries of procainamide (PR), tetraphenylborate (TPB), and their salt (procainamide with tetraphenylborate) were utilized. As a result of these interactions, several configurations of ion pairs were generated. One of the produced geometries was utilized in the same-level vibrational frequency computations to represent all stationary regions as minima (no imaginary frequencies) and to assess their thermodynamic features [42]. The proton affinities PR of procainamide and acid-conjugated bases were estimated by reversing the sign of the difference between the enthalpy values of the cations and acids and their respective tetraphenylborate (TPB) and base. The change in Gibbs free energy G_298_ associated with ion pair formation was calculated using the difference between the free energy of the ion pair and the sum of the free energies of the tetraphenylborate and procainamide ions (at the standard condition). The interaction energy E_int_ between the ions in the ion pair was computed using the super molecule technique [51,54], i.e., as the energy difference between the ion pair and its constituent ions. The Boys and Bernardi counterpoise methods [55] were used for the ion pairs’ optimized structures in order to calculate the basis set superposition error (BSSE). When the BSSE is considered, the resultant interaction energy is somewhat reduced (by no more than 2%), but the relative order of the energies in the sequence of examined compounds stays unchanged. The approach of Bader’s quantum theory of atoms in molecules (QTAIM) was utilized to investigate the topological electron density [38,56]. Numerous studies focused on clarifying noncovalent interactions have also utilized this technique [42,43]. Within the QTAIM paradigm, atomic interactions are intricately tied to the topological features of the electron density ρ(r), especially the set and kinds of critical sites at which its gradient is zero. The bond critical point (BCP) [47,49] and the bond path passing through it are crucial to our research since they are essential for the chemical bond or, more generally, for stabilizing the interatomic interaction between the two bound atoms. Consequently, the bigger the ρ(r) value at the BCP, the greater the electronic charge concentration in the surface at this point, and the greater the contact strength. Compared to covalent bonding, where ρ(r) is less than ∼ 10^–1^ au, the values of ρ(r) for hydrogen bonding [56] and van der Waals interactions are rather small: 10^–2^ au for hydrogen bonding [56] and 10^–3^ au for van der Waals interactions [57].

The ρ(r) value of interacting atoms at their BCP, as well as their Laplacian ∇2ρ(r), H(r), [56], and |V(r)|/G(r) [58], are the most commonly employed measures to characterize the nature and strength of bonding interactions. The following topological characteristics can be used to differentiate between three distinct types of bonding interactions: (i) 2∇^2^ρ (r) 0, H(r) 0, and |V(r)|/G(r) > 2 for shared interactions (covalent bonding); (ii) 2∇^2^ρ(r) > 0, H(r) > 0, and |V(r)|/G(r) 1 for closed-shell interactions (van der Waals interactions and weak electrostatic H-bonds); and (iii) 2∇^2^ρ(r) > (strong H-bonding). Koch and Popelier [39,40] presented two quantitative criteria for hydrogen bonding interactions inside the QTAIM; the ρ(r) and 2∇^2^ρ(r) at the BCP are between 0.002 and 0.035 au and 0.024 and 0.139 au, respectively. Utilizing ion-pair optimized geometries, all wave functions were calculated using the single-point approach. The QTAIM calculations were carried out using the AIMAll program (version 10.05.0483) [19]. The global and local indices were computed using the Koopman method [59,60,61]. To get the aforementioned indices, we conducted single-point calculations of the anion and cation at the ideal shape of the neutral molecule, while maintaining a constant external potential. The creation of new and more precise density functionals is a highly active area of study. GaussView 06 [62] was utilized to display the surface of the molecular electrostatic potential (MEP). Using Mulliken population analysis, the single-point energies of the “N, (N−1), and (N+1) species of the molecule were computed using the 6-311G (d, p) basis”. All DFT calculations were done at the ground state energy level of the 5-AU molecule, with no potential energy surface limitations.

## 4. Conclusions

In conclusion, the proposed ion-associate complex was prepared efficiently by the reaction of procainamide with sodium tetraphenyl borate in deionized water at an ambient temperature. The structure of the complex was characterized by different spectroscopic techniques, e.g., IR, NMR, and mass and elemental analysis, and their geometric structures and electrical characteristics were fully examined using experimental- and DFT-level theoretical calculations. Spectroscopic methods were used to characterize the structure (FT-IR and 1HNMR). In the ground state, DFT/B3LYP/6-311G(d,p) basis sets were used to determine the electrical and geometric properties of the S1 and S2 configurations of the title complex. Comparing the observed and calculated values for the S1 and S2 forms, the HOMO-LUMO energy gap of compounds was 3182 and 3231 eV, respectively. The small energy gap between HOMO and LUMO indicated that the compound was stable. In addition, the MEP reveals that positive potential sites were around the PR molecule, whereas negative potential sites were surrounding the TPB site of atoms. The UV absorption of both arrangements is comparable to the experimental UV spectrum.

## Figures and Tables

**Table 1:** Antimicrobial activity of the proposed compound.

	PR-TPB Compound		Imipenem		Fluconazole	
	Inhibition Zone (mm)	MIC (µg/mL)	Inhibition Zone (mm)	MIC (µg/mL)	Inhibition Zone (mm)	MIC (µg/mL)
*E. coli*	8	1024	30	≤0.25	ND	ND
*Ps. aeruginosa*	8	1024	28	0.5	ND	ND
*S. aureus*	14	64	29	≤0.25	ND	ND
*B. subtilis*	16	32	35	≤0.25	ND	ND
*Candida albicans*	15	64	ND	ND	26	2

ND = not determined; mm = millimeter; MIC = Minimum inhibitory concentration.

**Table 2:** Interaction energies with the (∆E)corrected and ∆EBSSE (kcal mol^−1^ )respectively, at the B3LYP/6-311G(d,p) level of theory.

Structure No.	Complexes		(∆E) Corrected (kcal/mol)	∆EBSSE
D	PR─TPB	–104.92	–99.13	0.00922
C	PR─TPB	–98.68	–94.64	0.00645

**Table 3:** Calculated energies, dipole moments (D), maximum absorption wavelengths (λ_max_), excitation energies (eV), oscillator strengths (*f*), assignment of electronic transitions (HOMO(H) → LUMO(L)), and major contribution (%) for the title compound S1 & S2 in gas phase gas.

Structure	Solvent	Parameters						
		E_Total_ (a.u)	Dipole Moment	λ_max_	*f*	Transition Energy (eV)	Electronic Transition	Major % Contribution
S1	Gas phase	–1699.89	21.08	312.51	0.006	3.9674	H-6 → L H-7 → L	96% 3%
	Water	–1699.87	25.06	278.20	0.5491	4.4566	H-2 → L H-1 → L H → L	15% 67% 14%
				237.11	0.0302	5.2290	H → L + 3 H → L + 4 H-4 → L + 2 H-2 → L + 3 H-1 → L + 4	26% 48% 3% 6% 2%
S2	Gas phase	–1699.89	19.56	297.35	0.0282	4.1695	H-7 → L H-6 → L H-5 → L	14% 24% 61%
	Water	–1699.88	23.45	283.10	0.537	4.3795	H-2 → L	94%
				248.41	0.102	4.9911	H-1→ L + 1 H-1→ L + 2 H-1→ L + 3	10% 48% 29%

**Table 4:** The most common experimental and calculated vibrational frequencies (cm^−1^) and assignments of the complex at ”B3LYP/6-311G(d,p)”.

Assignments	Experiment (cm^−1^)	Calculated (cm^−1^)			
		S1	Relative Error	S2	Relative Error
*υ*NH_2_	3230–3469	3589.56	120.56	3552.23	83.23
*υ*NH	2753	2954.52	201.52	2673.21	–79.79
*υ*C=N	1629	1672.26	43.26	1620.46	–8.54
*υ*C=O	1633	1657.54	24.54	1618.76	–14.24

**Table 5:** Experimental and theoretical (B3LYP/6-311 + G(d,p)) GIAO ^1^H chemical shift values (with respect to TMS, all values in ppm) of the proposed complex.

Atoms		Experimental Chemical Shift (ppm)	Calculated Chemical Shift (ppm)	
Type	Label Numbers		S1	S2
CH3	71,74,78,72,77,70	1.16	1.012	1.089
CH2	63,61,67,64,75,60,79,68	3.13	2.60899	2.22
NH2	82,83	3.53	3.79	3.42
NH	58	6.6	5.8	5.76
O-ArH	42,55,54	6.8	6.692	6.49
p-m-ArH	34,44,23,32,21,43,41,45,17	6.92	7.10783	6.96
ArH	8,19,11,22,33,10,28,12,53	7.15	7.43665	7.66
ArH	6,52,30	7.54	7.8617	8.26
H+	84	8.37	8.9439	─

**Table 6:** AIM parameters of chosen H-bonds at bond critical points (BCPs) for the interaction between procainamide and tetraphenyl borate complex.

BCP	Bond	ρ(r) (a.u.)	K(r) (a.u.)	V(r) (a.u.)	H(r) (a.u.)	∇^2^ρ(r) (a.u.)	G(r) (a.u.)	$\frac{\left|V\left(r\right)\right|}{G\left(r\right)}$	$\frac{H\left(r\right)\;}{\rho \left(r\right)}$
Complex 1 (S1)									
1	C2-H64	0.0049	–0.0006	–0.0022	0.0006	0.0139	0.0029	0.787	0.1241
19	H19-H64	0.0053	–0.0008	–0.0028	0.0008	0.0178	0.0036	0.7748	0.154
30	C25-H64	0.0076	–0.0008	–0.0032	0.0008	0.0194	0.004	0.7962	0.108
72	C4-H60	0.0073	–0.0007	–0.0034	0.0007	0.0195	0.0041	0.8212	0.1017
73	C5-H61	0.0061	–0.0007	–0.0028	0.0007	0.0172	0.0036	0.7945	0.1205
76	C3-H75	0.004	–0.0005	–0.0018	0.0005	0.0113	0.0023	0.7751	0.1298
77	C27-H67	0.0067	–0.0006	–0.0029	0.0006	0.0166	0.0035	0.8185	0.0955
90	C31-H74	0.0066	–0.0006	–0.003	0.0006	0.0169	0.0036	0.8323	0.0917
Complex 2 (S2)									
14	N12-C41	0.0037	–0.0005	–0.0019	0.0005	0.0114	0.0024	0.6565	0.1313
44	H8-H44	0.0038	–0.0009	–0.0015	0.0009	0.0129	0.0024	0.4565	0.2308
45	H18-C47	0.0093	–0.0011	–0.0047	0.0011	0.0279	0.0058	0.6728	0.1231
48	H26-C43	0.0059	–0.0009	–0.0025	0.0009	0.0175	0.0035	0.5819	0.1545
58	H15-C52	0.0087	–0.0011	–0.0043	0.0011	0.0262	0.0054	0.6591	0.1288
61	H8-H57	0.0043	–0.0009	–0.0017	0.0009	0.0141	0.0026	0.4827	0.2145
62	H13-C53	0.0129	–0.001	–0.0076	0.001	0.0384	0.0086	0.7912	0.0781

**Table 7:** Shows the calculated HOMO(N), HOMO (N + 1), HOMO (N-1) energy bands, chemical potential (𝜇), electronegativity (𝜒), global hardness (𝜂), global softness (𝑆), and global electrophilicity index (𝜔) for tetraphenylborate, procainamide (IE, in eV) and its complex.

Compounds	HOMO (N)	HOMO (N+1)	HOMO (N-1)	Vertical EA	Vertical IP	𝜒	𝜇	𝜂	𝑆	𝜔	N
PR	–4.972	2.324	–8.708	6.928	–0.633	3.148	–3.148	7.562	0.132	0.655	4.15
TPB	–5.72	–2.428	–9.468	7.505	3.731	5.618	–5.618	3.774	0.265	4.182	3.401
S1	–4.792	1.741	–7.947	6.195	–0.084	3.056	–3.056	6.279	0.159	0.744	4.329
S2	–4.547	1.861	–7.871	5.972	–0.293	2.84	–2.84	6.265	0.160	0.644	4.574

## Data Availability

All data in the manuscript are available by all authors.

## Supplementary Materials

The following supporting information can be downloaded at: https://www.mdpi.com/article/10.3390/molecules28052256/s1. Figure S1. 1H NMR of the proposed complex. Figure S2. 13C NMR of the proposed complex. Figure S3. IR spectrum of the proposed complex. Figure S4. Mass spectrum of the proposed complex, (A) Positive scan; (B) Negative scan.

## Sample Availability

Samples of the compounds are available from the authors.
