# Supplementary material for: Synthesis of 4-Amino-N-[2 (diethylamino)Ethyl]Benzamide Tetraphenylborate Ion-Associate Complex: Characterization, Antibacterial and Computational Study
Source: Molecules. 2023 Feb 28;28(5):2256. doi: 10.3390/molecules28052256 (PMC10005259; doi:10.3390/molecules28052256)
Supplement: Supplementary file 1 [file molecules-28-02256-s001.zip › molecules-2232985-supplementary.pdf]

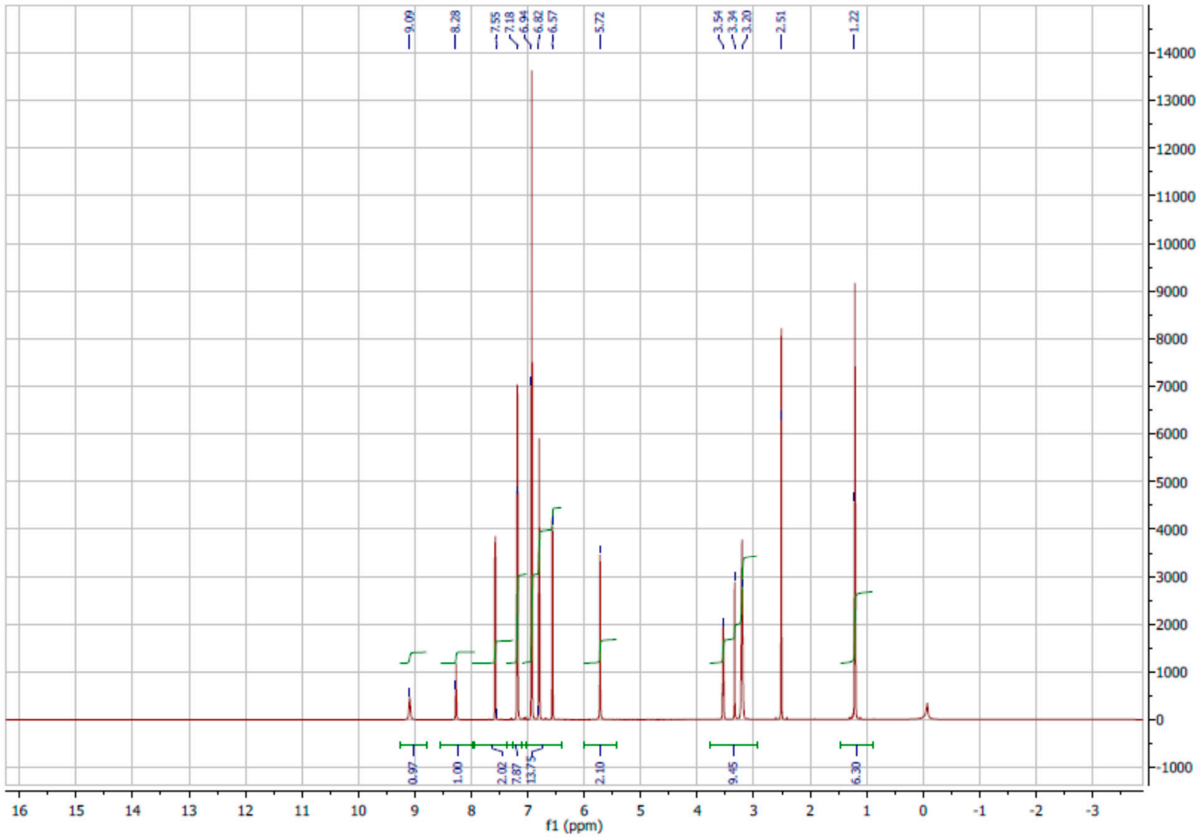

Figure S1. 1H NMR of the proposed complex.

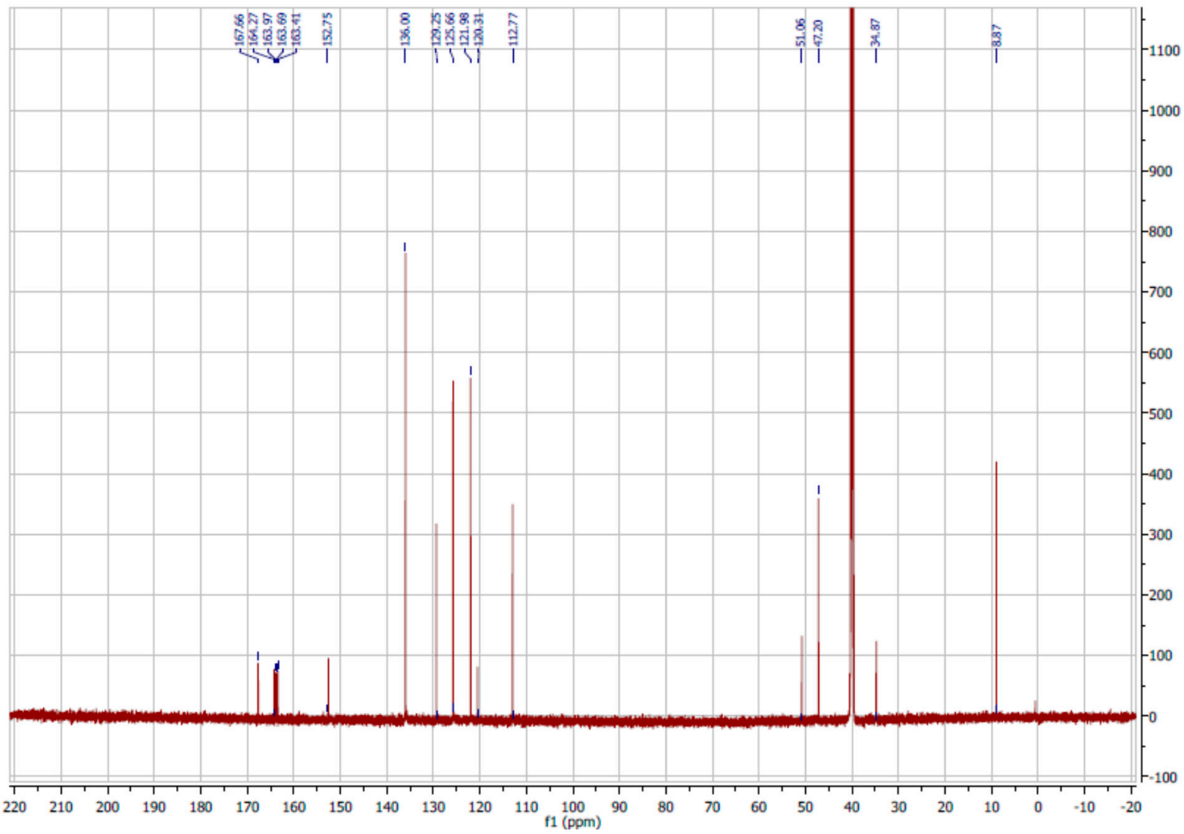

Figure S2. 13C NMR of the proposed complex

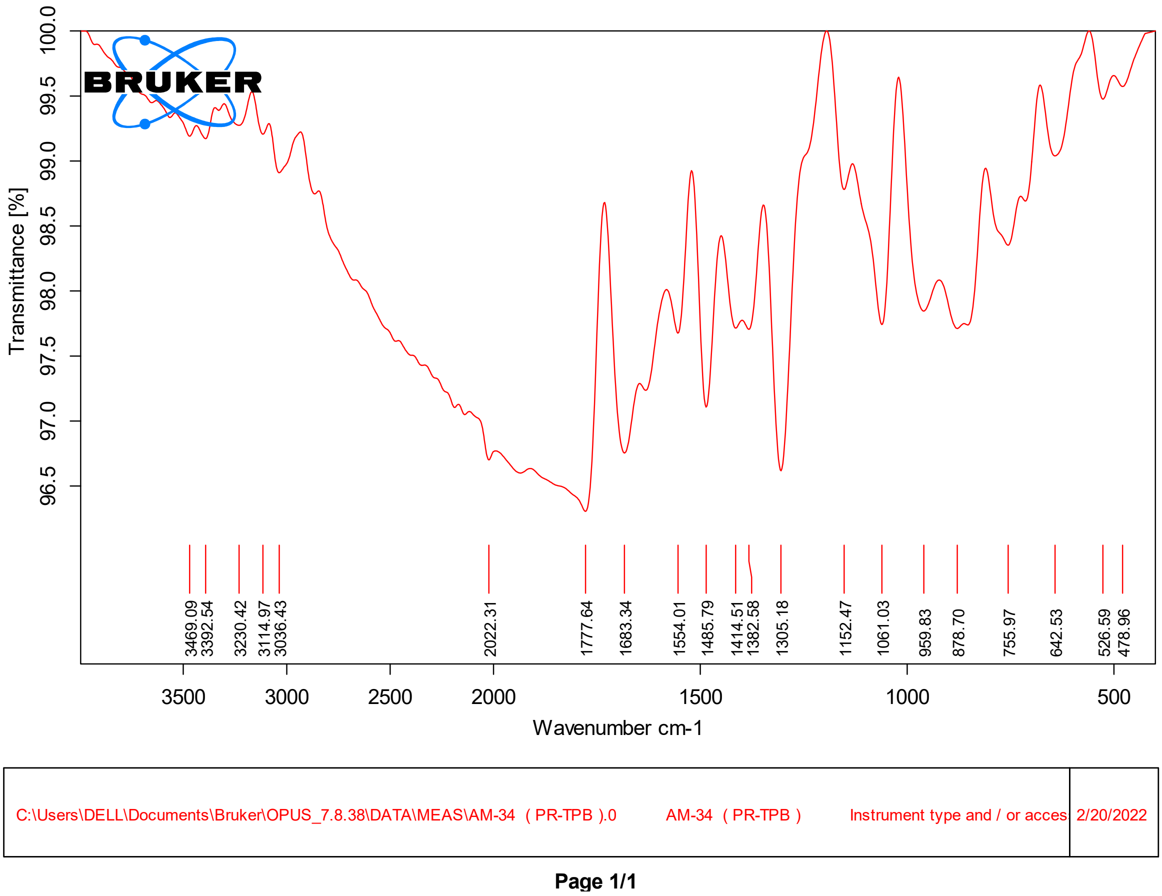

Figure S3. IR spectrum of the proposed complex.

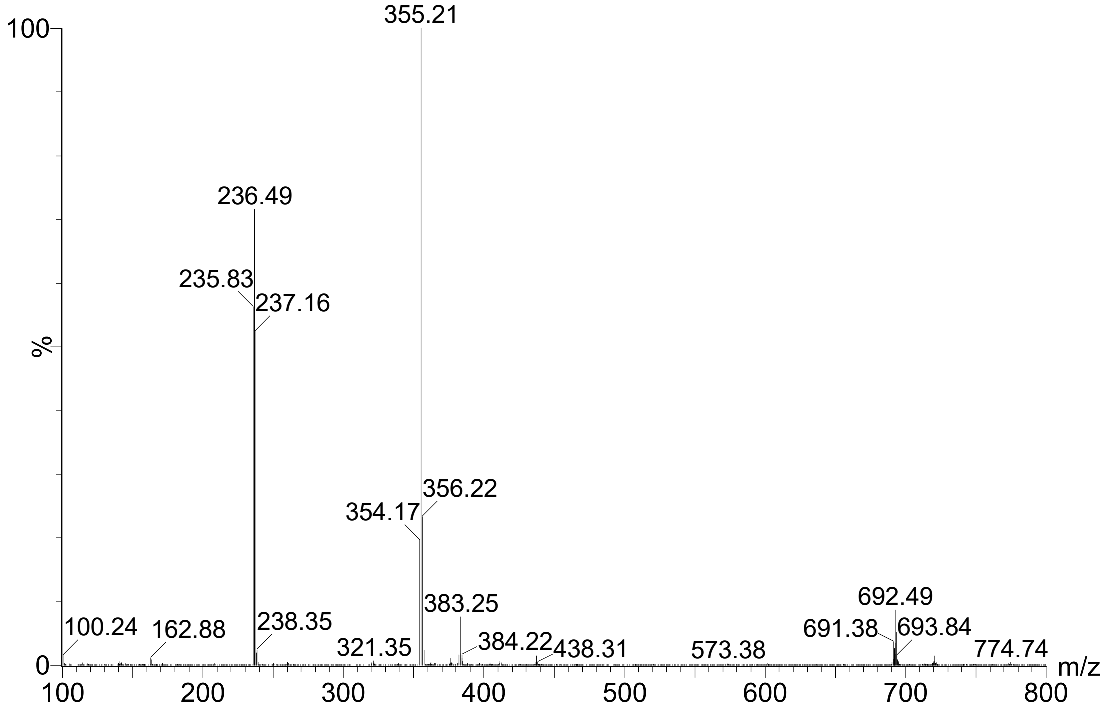

Figure S4A. Positive scan

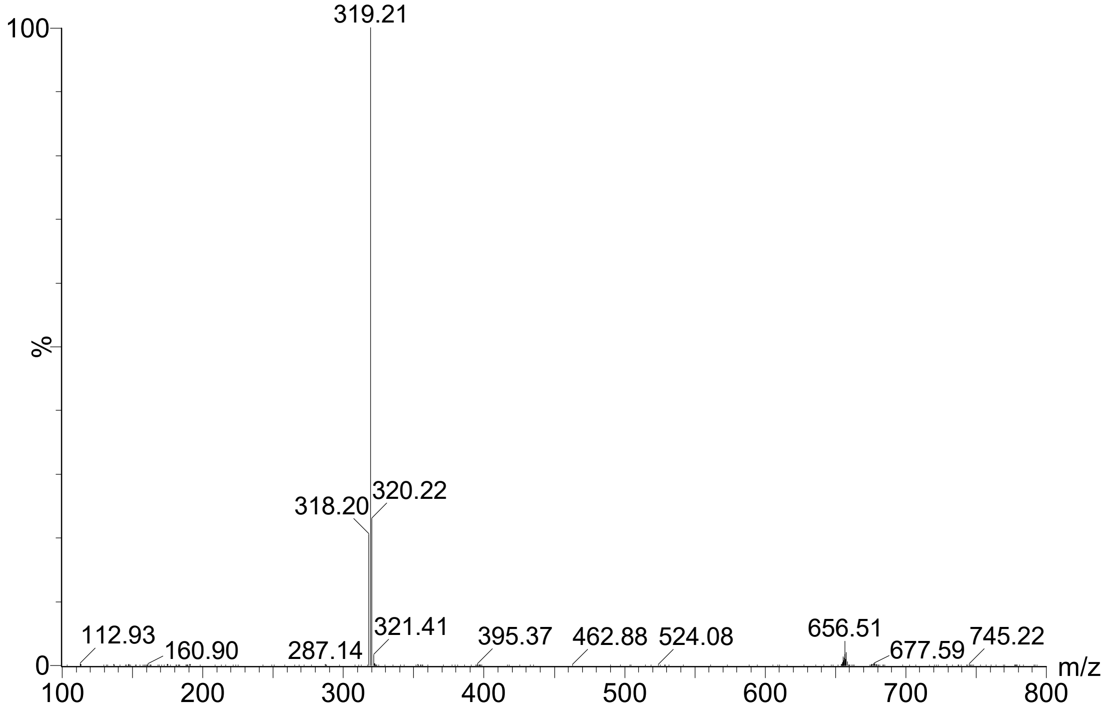

Figure S4B. Negative scan.

Figure S4. Mass spectrum of the proposed complex.
